# Discovery of Novel eEF2K Inhibitors Using HTS Fingerprint Generated from Predicted Profiling of Compound-Protein Interactions

**DOI:** 10.3390/medicines8050023

**Published:** 2021-05-20

**Authors:** Atsushi Yoshimori, Enzo Kawasaki, Ryuta Murakami, Chisato Kanai

**Affiliations:** 1 Institute for Theoretical Medicine, Inc., 26-1, Muraoka-Higashi 2-chome, Fujisawa 251-0012, Japan; yoshimori@itmol.com; 2 INTAGE Healthcare, Inc., 79, Kankoboko-cho, Shimogyo-ku, Kyoto 600-8009, Japan; kawasaki@intage.com (E.K.); murakami-r@intage.com (R.M.)

**Keywords:** eEF2K—Eukaryotic elongation factor-2 kinase, CGBFP—chemical genomics-based fingerprint, CGBVS—Chemical Genomics-Based Virtual Screening, CGBSP—Chemical Genomics-Based Similarity Profiling

## Abstract

**Background:** Eukaryotic elongation factor 2 kinase (eEF2K) regulates the elongation stage of protein synthesis by phosphorylating eEF2, a process related to various diseases including cancer and cardiovascular and neurodegenerative diseases. In this study, we describe the identification of novel eEF2K inhibitors using high-throughput screening fingerprints (HTSFP) generated from predicted profiling of compound-protein interactions (CPIs). **Methods:** We utilized computationally generated HTSFPs referred to as chemical genomics-based fingerprint (CGBFP). Generally, HTSFPs are generated from multiple biochemical or cell-based assay data. On the other hand, CGBFPs are generated from computational prediction of CPIs using the Chemical Genomics-Based Virtual Screening (CGBVS) method. Therefore, CGBFPs do not have missing information mainly caused by the absence of assay data. **Results:** Chemogenomics-Based Similarity Profiling (CGBSP) of the screening library (2.6 million compounds) yielded 27 compounds which were evaluated for in vitro eEF2K inhibitory activity. Three compounds with interesting results were identified. Compounds 2 (IC${}_{50}$ = 11.05 μM) and 4 (IC${}_{50}$ = 43.54 μM) are thieno[2,3-b]pyridine derivatives that have the same scaffolds with a known eEF2K inhibitor, while compound 13 (IC${}_{50}$ = 70.13 μM) was a new thiophene-2-amine-type eEF2K inhibitor. **Conclusions:** CGBSP supplied an efficient strategy in the identification of novel eEF2K inhibitors and provided useful scaffolds for optimization.

## 1. Introduction

Protein synthesis is a key process in living cells, being required for creating proteins through translation of mRNAs [1]. Eukaryotic elongation factor 2 (eEF2) is an essential factor for protein synthesis which mediates the movement of ribosomes along mRNAs from one codon to the next during the elongation stage of translation [2]. The activity of eEF2K is normally dependent on Ca${}^{2+}$ ions and calmodulin (CaM), which binds the N-terminal of its catalytic domain [2,3]. eEF2 is phosphorylated by Eukaryotic elongation factor 2 kinase (eEF2K) on Thr56. When eEF2 is phosphorylated, its affinity for ribosomes decreases [4,5]. Recent studies have revealed that eEF2K is associated with certain diseases including cancer [6] and cardiovascular [7] and neurodegenerative diseases [8]. eEF2K belongs to the α-kinase family, which is a small subgroup of atypical protein kinases displaying little sequence similarity to conventional protein kinases [9]. Unlike conventional protein kinases, eEF2K was only slightly inhibited by Staurosporine or its derivatives such as K252a or Goe6976 [10]. This feature makes the design of eEF2K inhibitors difficult, since it cannot be based on existing kinase inhibitors. Furthermore, the crystal structure of eEF2K has not been solved yet. Several eEF2K inhibitors with moderate activity have been reported [11,12,13,14,15,16]. A-484954 is a selective inhibitor with an IC${}_{50}$ value of 0.28 μM against eEF2K in enzymatic assay [12]. Thieno[2,3-b]pyridine analogues were reported as eEF2K inhibitors with sub-micromolar inhibitory activity [15]. Since no potent eEF2K inhibitor has been designed so far, new scaffolds are needed for optimization.

High-throughput screening fingerprint (HTSFP) was introduced by Petrone et al. [17] and is getting a lot of attention as a molecular representation that can be used for virtual screening [18], target identification [19], and hit expansion [20]. In HTSFP, each molecule is represented by hundreds of biochemical and cell-based HTS assay readouts, where a value or a bit in their fingerprint is set according to an assay readout [17,21]. PubChem HTS fingerprints were constructed from 243 bioassays of more than 300,000 compounds [21]. These 243 bioassays, which consist of 111 biochemical and 132 cell-based assays, were extracted from the PubChem BioAssay database [22]. Virtual screening using the PubChem HTSFP retrieved structurally diverse hits compared with traditional structural 2D fingerprint. HTSFP is agnostic with regard to chemical structures since it is based on bioassay data. Therefore, it has a high potential to accomplish scaffold hopping and identify active compounds [21]. Cheng et al. used 60 bioassays (referred to as NCI-60) taken from 73 NCI human tumor cell line growth inhibition assays of the PubChem BioAssay database, to construct bioactivity profiles for 4296 small molecules and predict compound-target associations [23]. In the process of constructing the bioactivity profiles, compounds with missing log (GI${}_{50}$) value in one or more of the NCI-60 cell lines were discarded, where GI${}_{50}$ is the concentration required for the 50% growth inhibition of tumor cells. Cabrera et al. identified novel nanomolar inhibitors of cellular division that reproduce the phenotype, and their study showed that HTFSPs are valuable tools for scaffold hopping [24]. Despite this success, HTSFP has one major limitation. The generation of HTSFP can only be made for previously tested compounds in HTS assays. Herein, we propose chemical genomics-based fingerprints (CGBFP) that can be generated from computational prediction of compound-protein interactions (CPIs) with which we have identified novel eEF2K inhibitors. The CGBFP of a compound is built on predicted activities against 599 target proteins using Chemical Genomics Based Virtual Screening (CGBVS) technique [25,26]. Generation of CGBFP can be made for all compounds because it is, basically, computationally generated HTSFP.

In our exploration of novel eEF2K inhibitors, we performed a technique called Chemical Genomics-Based Similarity Profiling (CGBSP), which is a technique that utilizes CGBFPs. This led to the identification of 27 compounds from the Enamine screening library. These compounds were then evaluated using eEF2K inhibitory activity assays. Among them, we found three novel eEF2K inhibitors.

## 2. Materials and Methods

### 2.1. Compounds

All tested compounds were purchased from Namiki Shoji Co., Ltd. (Tokyo, Japan).

### 2.2. CGBFP

CGBFPs were generated from the output of predictive models of CGBVS, which is represented as a vector of length 599, where each component encodes the binding probability score of the predictive model for a target protein. Details of the 599 target proteins are shown in Table S1 (Supplementary Data). CGBVS was developed by Yabuuchi et al. [25] to predict CPIs. In this study, CGBFPs were generated by CGBVS as implemented in the CzeekS software package [27]. CzeekS has 6 predictive models that correspond to 1190 target proteins. During the generation of CGBFPs, only target proteins trained with 100 or more CPI datasets were used, and this led to screening against 599 out of the available 1190 target proteins.

### 2.3. Virtual Screening Using CGBSP

CGBSP was performed by generating CGBFPs of test and reference compounds followed by calculation of cosine similarity [28]. Twenty eEF2K inhibitors obtained from ChEMBL [29] (Supplementary Data in Table S2) were used as reference compounds, and approximately 2.6 million compounds from the Enamine screening library were screened. 1-nearest neighbor (1-NN) similarity search method was applied to screen the database. 1-NN similarity search is a method to select compounds from the database that are most similar to one of the reference structures [30]. CGBFPs of the reference and the database compounds were calculated by CzeekS, and cosine similarity was used as the metric of similarity between the CGBFPs. After virtual screening, the top 40 scoring compounds were selected as candidate eEF2K inhibitors. Finally, 27 purchasable compounds (see Supplementary Data in Table S3) among them were evaluated by assay.

### 2.4. *In Vitro* eEF2K Assay

Off-Chip Mobility Shift Assay (MSA) for the inhibitory activities of the compounds against eEF2K was conducted by Carna Biosciences, Inc. (Kobe, Japan) using QuickScout Screening Assist™ Mobility Shift Assay Kit. A (4×) test substance solution was added to the assay buffer (20 mM HEPES, 0.01% Triton X-100, 1 mM DTT, pH 7.5). A (4×) substrate/ATP/metal solution was prepared using Kit buffer (20 mM HEPES, 0.01% Triton X-100, 5 mM DTT, pH 7.5). A (2×) kinase solution was prepared in assay buffer. Five microliters of 4× test substance solution, 5 μL of prepared substrate/ATP/metal solution, and 10 μL of kinase solution were mixed in a polypropylene 384-well plate and incubated at room temperature for 5 h. The reaction was stopped by adding 70 μL of Termination Buffer (QuickScout Screening Assist MSA; Carna Biosciences). Substrate and phosphorylated peptides in the reaction solution were separated and quantified by the LabChip™ system (Perkin Elmer). The kinase activity was evaluated as the product ratio (P/(P+S)) calculated from the peak height of the substrate peptide (S) and the peak height of the phosphopeptide (P). The average signal in the control wells containing all reaction components was set to 0% inhibition, and the average signal in the background wells (without enzymes) was set to 100% inhibition. Then the percentage of inhibition was calculated from the average signal of each test well. Inhibitory activity of the 3 inhibitors against 3 other calmodulin-related kinases (Calcium/calmodulin-dependent protein kinase 4 or CaMK4, Checkpoint kinase 1 or CHK1, Death-associated protein kinase 1 or DAPK1) were measured at 30 μM concentration. The IC${}_{50}$ values against eEF2K were determined from the plotted concentration of test compounds against the inhibition rate by approximating the logistic curve using the non-linear least squares for four parameters analysis as implemented in GraphPad Prism version 9.0.2 [31] for Windows.

### 2.5. Molecular Docking

eEF2K homology model was constructed using the SWISS-MODEL [32] server with Myosin heavy-chain kinase A crystal structure (PDB: 4ZS4 [33]) as template. AutoDock Vina [34] as implemented in YASARA [35] was used to predict binding modes of the three hit compounds. Docking was performed using the macro file dock_run.mcr with default parameters. The binding configuration of ATP to eEF2K was constructed by superimposing the crystal structure of ATP and myosin heavy chain kinase A (PDB: 4ZS4) complex on the eEF2K homology model.

## 3. Results

### 3.1. Concept of CGBFP

**Figure 1:**
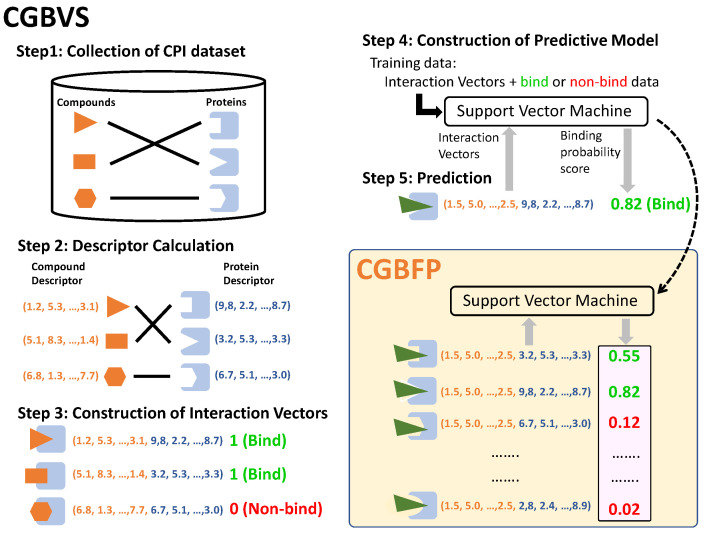
Workflow of CGBVS and CGBFP generation. The CGBVS section illustrates construction of predictive models for compound-protein interactions (CPIs) via Step 1 to Step 5. The CGBFP section shows the generation of CGBFP using CGBVS predictive models (Support Vector Machine). Values within the pink box represent CGBFPs.

CGBFPs are, basically, HTSFPs computationally generated from predicted profiling of CPIs. It consist of binding probability scores obtained from predictive models of CGBVS [25,26] (Figure 1). CGBVS is a machine-learning-based method for predicting the binding probability score of a compound based on the binding patterns obtained from the interaction information (chemical genomics information) between the protein (biological space) and the compound (chemical space). The steps leading to the construction of predictive models are illustrated in Figure 1. CPI datasets were obtained from ChEMBL release 25 (Step 1). Compound descriptors were generated using alvaDesc [36] software, which is the successor to the widely used DRAGON [37] software. Protein descriptors were calculated using the PROFEAT 2016 web server [38] (Step 2). Interaction vectors were constructed by combining compound and protein descriptors (Step 3). Concatenated vectors for CPI (binding) pairs and noninteracting (non-binding) pairs were used as input into support vector machine (SVM) (Step 4). Binding probability scores of target proteins were obtained from the SVM. Scores are values between 0 and 1, and scores greater than or equal to 0.5 are considered positive or indicate potential binding between the test compound and target protein (Step 5).

### 3.2. Virtual Screening Using CGBSP

As already stated, successful examples of virtual screening using HTSFP have been reported [18,19,20] previously. In this study, we performed virtual screening based on CGBFP.

The use of CGBFP has advantages as follows:

(1) CGBFPs are computationally generated; therefore, it has no missing information principally caused by the absence of assay data usually seen with HTSFP.
(2) Generation of CGBFPs can be performed for all compounds, in contrast to HTSFP, which can only be performed for previously tested compounds in HTS assays.

**Figure 2:**
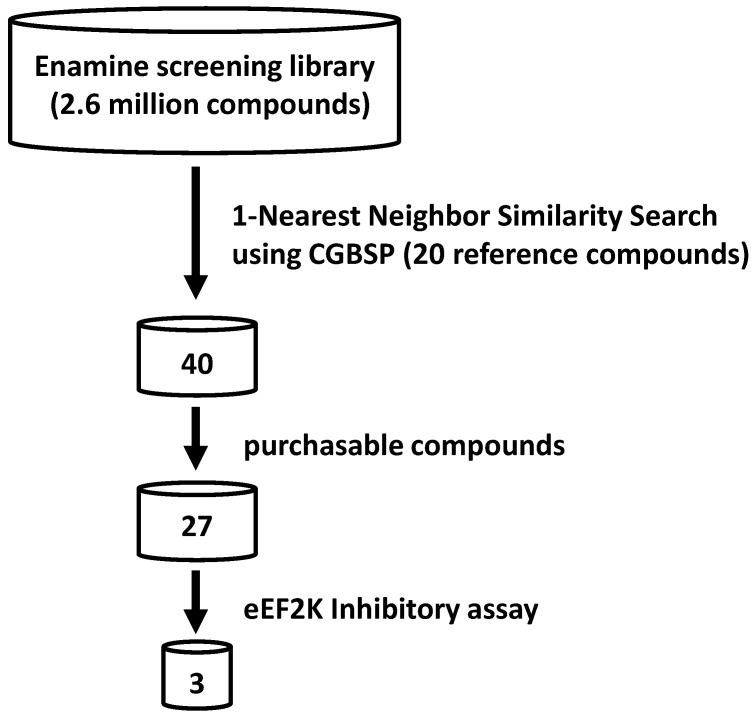
Overview of virtual screening using CGBSP.

Figure 2 illustrates the workflow of our virtual screening protocol. In our efforts to identify novel eEF2K inhibitors, we virtually screened the Enamine screening library comprising approximately 2.6 million compounds. 1-NN similarity search was conducted based on twenty reference compounds (known eEF2K inhibitors; see Supplementary Data in Table S2) using CGBFP.

Representative eEF2K inhibitors from the reference compounds are listed in Table 1. The top 40 scoring compounds from the 1-NN similarity search were initially selected, from which 27 compounds were further selected and then purchased in order to perform the biological assays.

### 3.3. Enzyme Inhibition Assays

**Figure 3:**
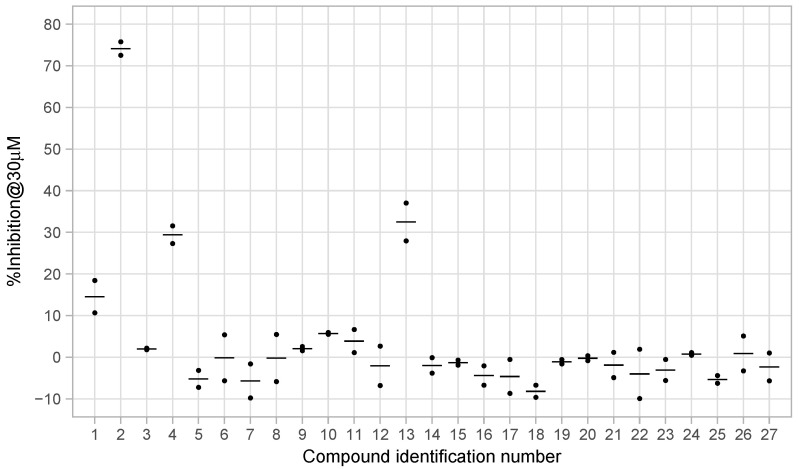
Preliminary measurements of eEF2K inhibitory activities of selected 27 compounds. Black dots indicate values for duplicate measurements and crossbars indicate mean values for each compound.

**Figure 4:**
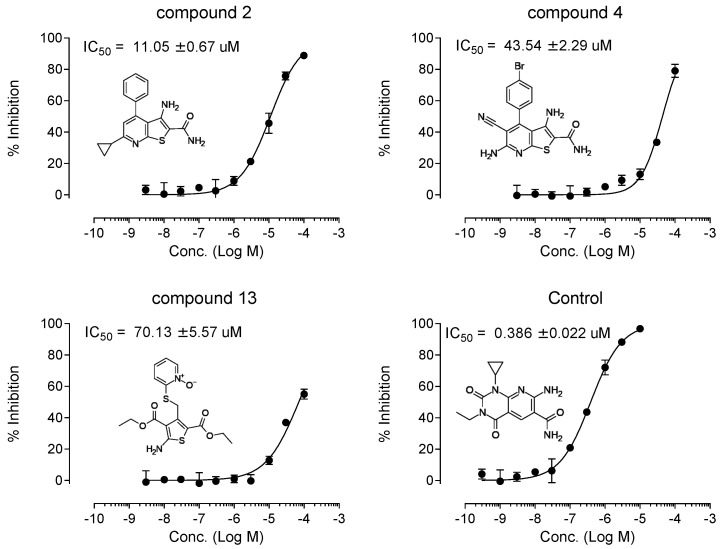
Dose-dependence curve of three hit compounds and A-484954 as control. The compound concentration required for 50% inhibition (IC${}_{50}$) was determined from semi-logarithmic dose–response plots. Values are mean of 4 replicates.

From the initial testing of 27 compounds for eEF2K inhibitory activities, three compounds (compounds 2, 4, and 13) showed over 25% inhibition (74.1%, 29.4%, and 32.5%, respectively) of eEF2K at the concentration of 30 μM (Figure 3). Further testing by dose–response assays showed compounds 2, 4, and 13 exhibiting good dose-dependent inhibitory effect with IC${}_{50}$ values of 11.05 ± 0.67, 43.54 ± 2.29, and 70.13 ± 5.57 μM, respectively, (Figure 4). In particular, compound 2 was shown to be a moderately effective inhibitor against eEF2K. A-484954 was used as the reference compound, and the calculated IC${}_{50}$ value is 0.386 ± 0.022 μM. This value was found to be in good agreement with previously reported value of 0.28 μM [12] (Table 1).

Compounds 2 and 4 are thieno[2,3-b]pyridine derivatives with the same scaffold with known eEF2K inhibitors (CHEMBL1094018 and CHEMBL1092820 in Table 1). We have found that the CGBSP approach could select compounds that have the same scaffolds as the reference compounds. On the other hand, compound 13 was identified as a new thiophene-2-amine type inhibitor of eEF2K. This scaffold is different with known scaffolds of eEF2K inhibitors (Table 1 and Supplementary Data in Table S2). CGBSP used the predicted bioactivity-based molecular descriptor named as CGBFP instead of the structural molecular descriptor such as extended connectivity fingerprints [39]. Therefore, CGBSP may have an ability to hop in chemical space of known scaffolds and identify active compounds. This result shows that CGBSP can be similarly used as a tool for scaffold hopping just like HTSFP.

### 3.4. Comparison of CGBFP Profiles between Reference and Hit Compound

**Figure 5:**
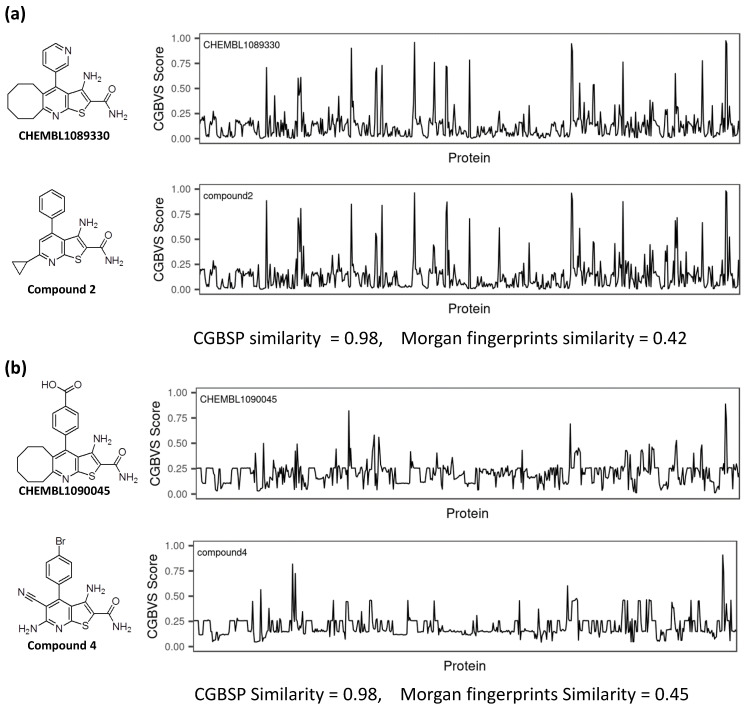

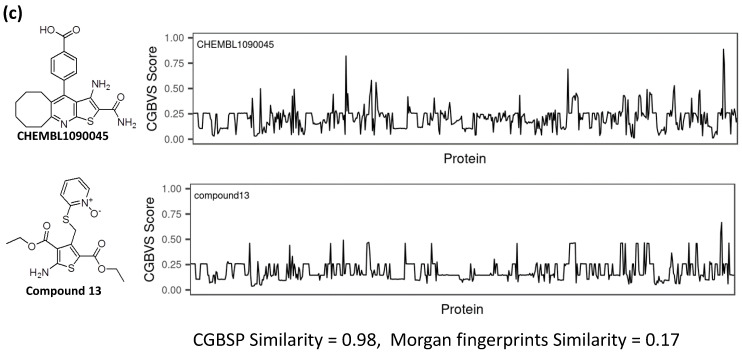
Comparison of CGBFP profiles between hit compound and its most similar (nearest neighbor) compound. The x-axis is represented by 599 CGBVS target proteins, and the y-axis indicates CGBVS score (binding probability score). CGBSP score is determined by calculating the cosine similarity between CGBFP of the hit compound and its most similar compound. Morgan fingerprint similarity is calculated by Tanimoto coefficient [40] between Morgan fingerprints of a compound and its most similar compound. The Morgan fingerprints were calculated with a radius of r = 2 and 2048 bit length using RDKit. (**a**) CHEMBL1089330 vs. Compound 2, (**b**) CHEMBL1090045 vs. Compound 4. (**c**) CHEMBL1090045 vs. Compound 13.

In this study, we identified three hit compounds using CGBFP, revealing its capability for virtual screening. Figure 5 shows the CGBFP profiles of the hit and the most similar reference (nearest neighbor) compounds, respectively. The CGBSP score of compound 2 against CHEMBL1089330 is high (0.98) while also giving a high Morgan fingerprint similarity score (0.42) (Figure 5a).

Generation of Morgan fingerprints, which is implemented in RDKit as an analogue of extended connectivity fingerprints [39], is a method of encoding a molecular structure. Means of Morgan fingerprint similarities of CHEMBL1089330 and CHEMBL1090045 against Enamine screening library were 0.1351 and 0.1446, respectively, (Figure S1). In the case of compound 4, the CGBSP score is 0.98 and the Morgan fingerprint similarity score is 0.45 (Figure 5b). Because compounds 2 and 4 have the same scaffolds (thieno[2,3-b]pyridine) with their reference compound, their Morgan fingerprint similarities are high. On the other hand, the CGBSP score of compound 13 is high (0.98), in contrast with Morgan fingerprint similarity score, which is moderate (0.17) (Figure 5c). This result shows that CGBSP enabled us to find hit compounds among structurally dissimilar molecules.

### 3.5. Molecular Docking Study

**Figure 6:**
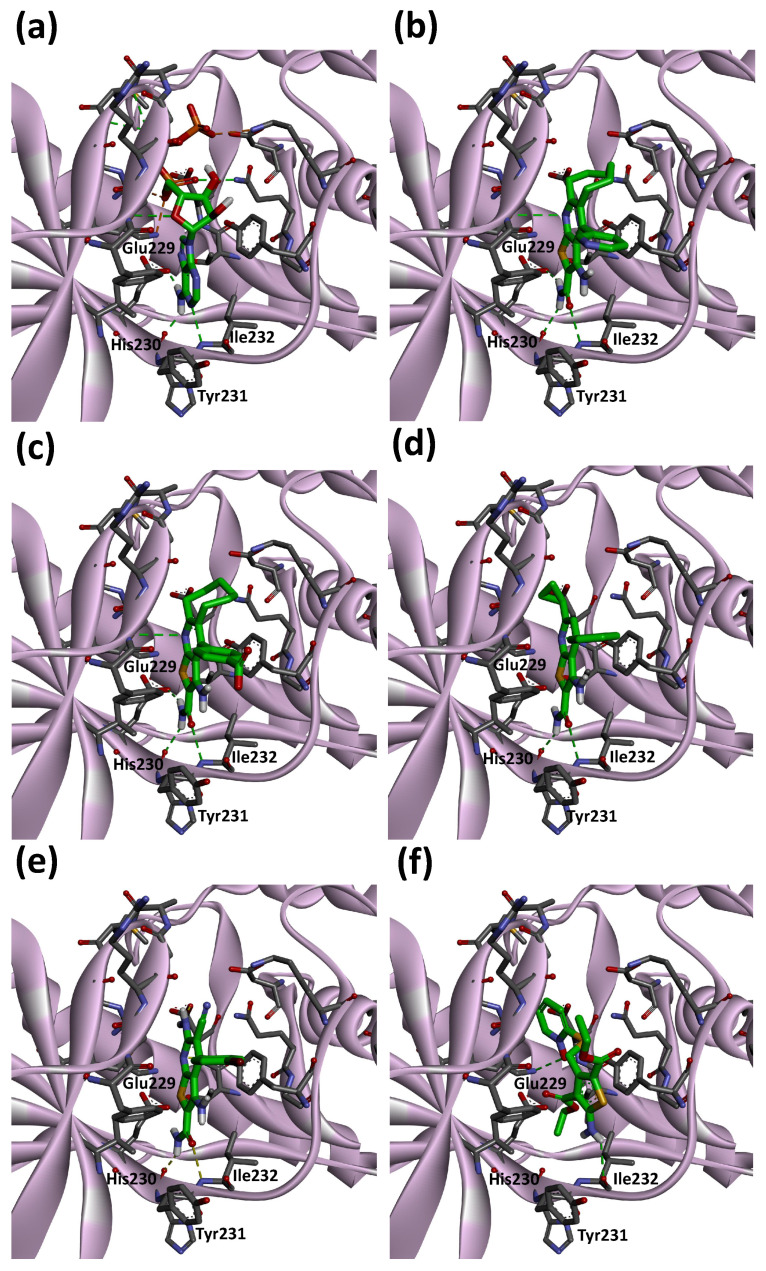
Predicted binding modes of ATP (**a**), CHEMBL1089330 (**b**), CHEMBL1090045 (**c**), compound 2 (**d**), compound 4 (**e**) and compound 13 (**f**) on eEF2K homology model. Carbon, nitrogen, oxygen and hydrogen are shown in green (ATP, compound 2, compound 4 and compound 13)/gray (eEF2K), blue, red and white, respectively. Dashed lines indicate hydrogen bond interactions. eEF2K is shown as ribbon representation.

To examine the binding modes of hit compounds against eEF2K, we performed a molecular docking study using AutoDock Vina [34] as implemented in YASARA [35] software. The structure of eEF2K was modeled by comparative protein modeling methods using the SWISS-MODEL server [32]. As shown in Figure 6a, ATP binds to the hinge region of eEF2K, which includes Glu229, His230, Tyr231 and Ile232 residues. The main chain amino group of Ile232 makes a hydrogen bond with the N1 of the adenine base and allows the carbonyl group of His230 and the side-chain of Glu229 to form hydrogen bonds with N6 of the adenine base. Since the majority of kinase inhibitors interact with a hinge region in the ATP binding sites of kinases, the interaction with hinge region of eEF2K is important in investigating the binding modes of hit compounds.

The binding modes of reference and hit compounds with hydrogen bonds with the hinge region are shown in Figure 6. Both amido groups of the reference compounds (CHEMBL1089330 and CHEMBL1090045) interact with the carbonyl group of His230 and amino group of Ile232 (Figure 6b,c). Similarly, the amido group of compound 2 forms hydrogen bond interactions with the carbonyl group of His230 and amino group of Ile232 (Figure 6d). The same type of interactions occur at the hinge region with compound 4 (Figure 6e). These two hydrogen bonds can be observed in ATP/eEF2K complex model (Figure 6a). On the other hand, the amino group of compound 13 forms a hydrogen bond with the carbonyl group of Ile232 (Figure 6f). This hydrogen bond interaction of compound 13 is unique for binding the hinge region of eEF2K. Although eEF2K inhibitory activity of compound 13 is not potent enough, the binding mode may have important implications for the modification of hit compounds obtained in this study.

## 4. Discussion

eEF2K belongs to a small subgroup of atypical protein kinases termed α-kinases. Up to the present, six human α-kinases have been identified. In addition to eEF2K, the human genome encodes α-kinases 1, α-kinases 2, α-kinase 3, TRPM6 and TRPM7. The α-kinases found in humans are widely distributed among vertebrates [9]. In contrast to other human α-kinases, eEF2K can also be found in invertebrates such as the metazoan *Trichoplax adhaerens* and in the diatom *Thalassiosira pseudonana*. According to the phylogenetic tree, eEF2K appears to be most closely related to the *Dictyostelium discoideum* MHCKs [9]. Design of inhibitors for eEF2K is difficult, because eEF2K displays little sequence similarity to conventional kinases, and so far, no inhibitor with nanomolar potency has yet been found. In the present work, we proposed the use of the CGBSP technique utilizing computationally generated HTSFP, which we refer to as CGBFP. CGBSP enabled us to identify three novel inhibitors of eEF2K. Two of them, compounds 2 and 4, have the same thieno[2,3-b]pyridine scaffold with known eEF2K inhibitors. Docking study results show that these inhibitors interact with the carbonyl group of His230 and the amino group of Ile232 on the hinge region of eEF2K by forming hydrogen bond interactions. The other compound (compound 13) is a new thiophene-2-amine-type eEF2K inhibitor. The results indicate that CGBSP contributed to bringing about scaffold hopping. Compound 13 interacts with the carbonyl group of Ile232 on the hinge region, which is a unique binding pattern to eEF2K. These results can be an indication that inhibitors that can interact with the three sites (carbonyl group of His230, amino group of Ile232 and carbonyl group of Ile232) may have a potent inhibitory activity against eEF2K. Selectivity of kinase inhibitor is a major challenge in drug design. eEF2K is also known as calcium/calmodulin-dependent eukaryotic elongation factor 2 kinase, which is activated by Ca${}^{2+}$ ions via CaM. To investigate selectivity of the three inhibitors against other CaM related kinases (CaMK4, CHK1 and DAPK1), inhibitory assays were performed (Figure S2). Compounds 2 and 13 slightly inhibited the activity of CaMK4 by 26.5% and 25.5%, respectively, at 30 μM. Compound 4 showed no significant inhibitory activity (10.8% at 30 μM) against CaMK4. The three inhibitors were observed to have no significant inhibitory activities (11.5% for compound 2, 5.0% for compound 4 and −0.1% for compound 13 at 30 μM) against CHK1. Compounsd 2 and 4 showed no significant inhibitory activities (16.1% and 15.9%, respectively, at 30 μM) against DAPK1, although compound 13 inhibited activity of DAPK1 by 37.5% inhibition at 30 μM. The results indicate the three inhibitors of eEF2K had some degree of selectivity over the CaM related kinases. Thus, the three inhibitors are considered to be useful leads for the design of selective eEF2K inhibitor and may also become useful tools for studying structure-activity relationship of eEF2K inhibitors.

## Figures and Tables

**Table 1:** Representative eEF2K inhibitors with the corresponding IC${}_{50}$ values obtained from relevant references.

ChEMBL ID ${}^{a}$	Structure	IC${}_{50}$ (M)	Ref
CHEMBL1094018	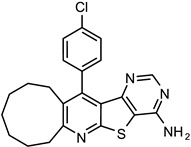	0.11	14
CHEMBL1092820	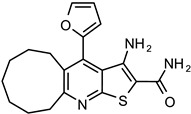	0.17	14
CHEMBL1977874 ${}^{b}$	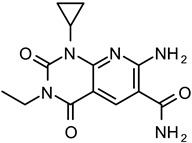	0.28	11

^a^ ChEMBL ID is the compound identification number in the ChEMBL database. ^b^ Refers to the eEF2K inhibitor A-484954.

## Data Availability

Not applicable.

## Supplementary Materials

The following are available online at https://www.mdpi.com/article/10.3390/medicines8050023/s1, Figure S1: Histogram of similarity between Enamine library (2.6 million) and reference compounds, Figure S2: Inhibitory activity of the 3 inhibitors identified in this study against calmodulin-related kinases, Table S1: List of target proteins included in the generation of CGBVS profiles, Table S2: List of reference compounds used in CGBSP, Table S3: List of compounds tested for in vitro inhibitory activity
