# Supplementary material for: Discovery of Novel eEF2K Inhibitors Using HTS Fingerprint Generated from Predicted Profiling of Compound-Protein Interactions
Source: Medicines (Basel). 2021 May 20;8(5):23. doi: 10.3390/medicines8050023 (PMC8161098; doi:10.3390/medicines8050023)
Supplement: Supplementary file 1 [file medicines-08-00023-s001.zip › medicines-1178481-supplementary.pdf]

Supplementary Materials: Discovery of Novel eEF2K Inhibitors Using HTS Fingerprint Generated from Predicted Profiling of Compound–Protein Interactions

Atsushi Yoshimori, Enzo Kawasaki, Ryuta Murakami and Chisato Kanai

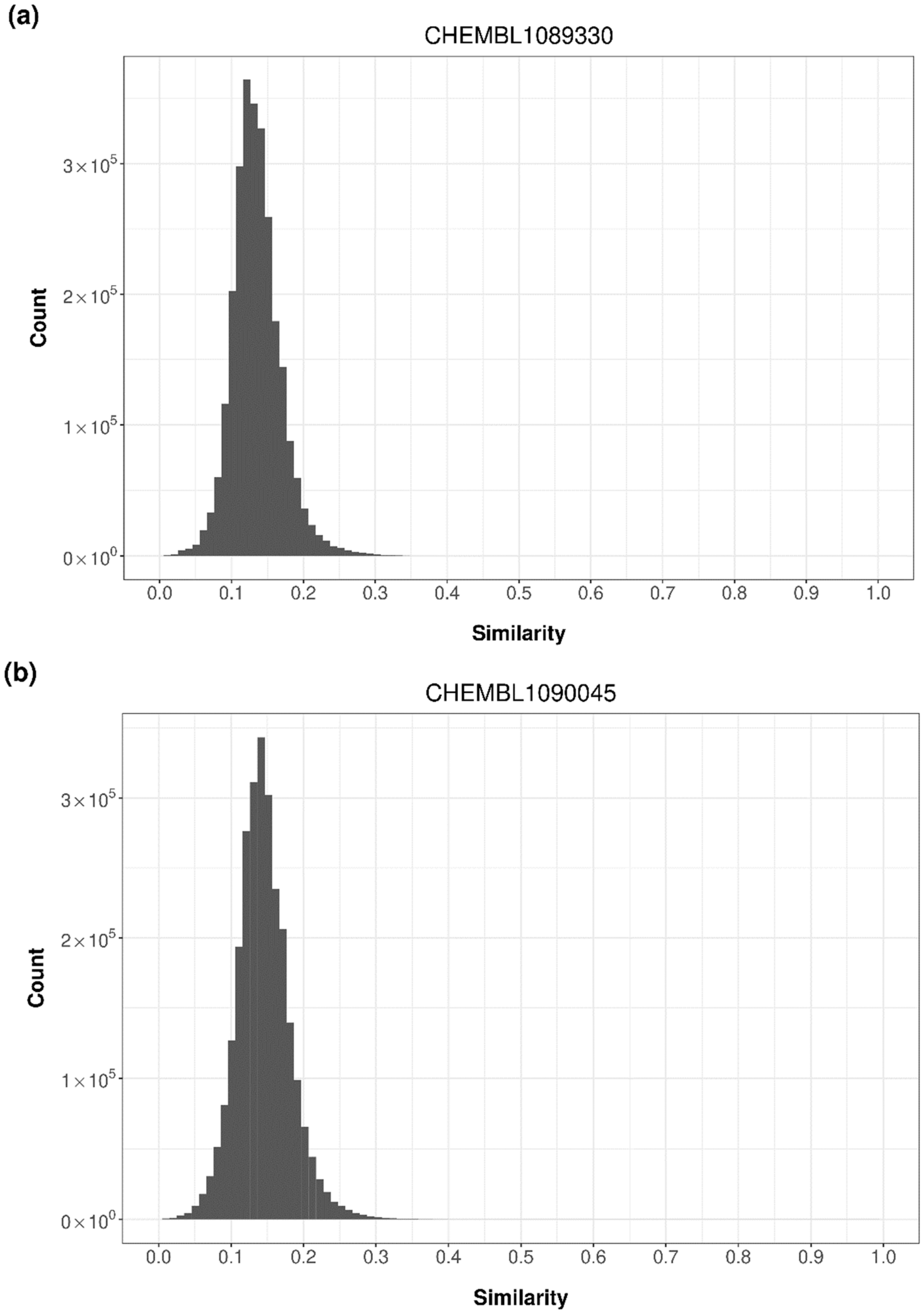

Figure S1. Histogram of similarity between Enamine library (2.6 million) and reference compounds. Similarities are calculated by Tanimoto coefficient with Morgan fingerprints (radius = 2 and bit lengh = 2048) using RDKit software. Histogram (**a**) is for reference compound CHEMBL1089330 and (**b**) is for reference compound CHEMBL1090045.

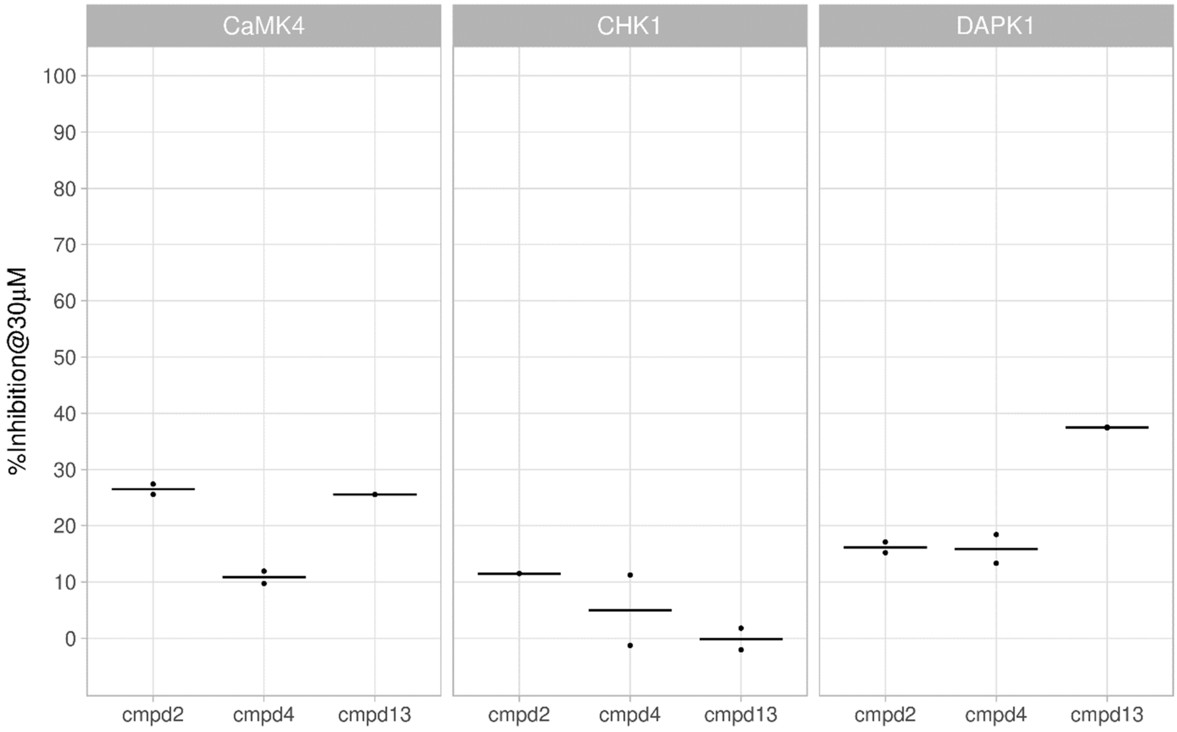

Figure S2. Inhibitory activities of the 3 inhibitors identified in this study against calmodulin-related kinases.

Table S1. List of target proteins included in the generation of CGBFP.

ID	Protein Name	UniProt Entry Name
1	Adenosine receptor A2a	AA2AR HUMAN
2	Adenosine receptor A1	AA1R HUMAN
3	Adenosine receptor A2b	AA2BR HUMAN
4	Activated CDC42 kinase 1	ACK1 HUMAN
5	Aurora kinase A	AURKA HUMAN
6	Aurora kinase B	AURKB HUMAN
7	Beta-secretase 1	BACE1 HUMAN
8	Beta-secretase 2	BACE2 HUMAN
9	B1 bradykinin receptor	BKRB1 HUMAN
10	B2 bradykinin receptor	BKRB2 HUMAN
11	Serine/threonine-protein kinase B-raf	BRAF HUMAN
12	Cathepsin S	CATS HUMAN
13	C-C chemokine receptor type 5	CCR5 HUMAN
14	Cyclin-dependent kinase 9	CDK9 HUMAN
15	Macrophage colony-stimulating factor 1 receptor	CSF1R HUMAN
16	Ephrin type-A receptor 2	EPHA2 HUMAN
17	Tyrosine-protein kinase Fer	FER HUMAN
18	Gamma-aminobutyric acid receptor subunit alpha-1	GBRA1 HUMAN
19	Glucagon receptor	GLR HUMAN
20	G-protein coupled receptor 55	GPR55 HUMAN
21	G-protein coupled receptor 6	GPR6 HUMAN
22	Insulin-degrading enzyme	IDE HUMAN
23	Insulin-like growth factor 1 receptor	IGF1R HUMAN
24	Potassium voltage-gated channel subfamily A member 3	KCNA3 HUMAN
25	Potassium voltage-gated channel subfamily A member 5	KCNA5 HUMAN
26	Mast/stem cell growth factor receptor Kit	KIT HUMAN
27	Tyrosine-protein kinase SYK	KSYK HUMAN
28	Leukocyte tyrosine kinase receptor	LTK HUMAN
29	Mitogen-activated protein kinase kinase kinase 12	M3K12 HUMAN
30	Mitogen-activated protein kinase kinase kinase 14	M3K14 HUMAN
31	MAP kinase-activated protein kinase 2	MAPK2 HUMAN
32	Melanocortin receptor 3	MC3R HUMAN
33	Melanocortin receptor 4	MC4R HUMAN
34	Melanocortin receptor 5	MC5R HUMAN
35	Mucolipin-3	MCLN3 HUMAN
36	Matrilysin	MMP7 HUMAN
37	MAP kinase-interacting serine/threonine-protein kinase 1	MKNK1 HUMAN
38	MAP kinase-interacting serine/threonine-protein kinase 2	MKNK2 HUMAN
39	72 kDa type IV collagenase	MMP2 HUMAN
40	Stromelysin-1	MMP3 HUMAN
41	Melatonin receptor type 1A	MTR1A HUMAN
42	Platelet-derived growth factor receptor alpha	PGFRA HUMAN
43	Progesterone receptor	PRGR HUMAN
44	DNA-dependent protein kinase catalytic subunit	PRKDC HUMAN
45	cAMP-dependent protein kinase catalytic subunit PRKX	PRKX HUMAN
46	Proteasome subunit beta type-5	PSB5 HUMAN
47	Presenilin-1	PSN1 HUMAN
48	Retinoic acid receptor gamma	RARG HUMAN
49	Macrophage-stimulating protein receptor	RON HUMAN
50	Retinoic acid receptor RXR-alpha	RXRA HUMAN
51	Retinoic acid receptor RXR-beta	RXRB HUMAN

ID	Protein Name	UniProt Entry Name
52	Sodium-dependent noradrenaline transporter	SC6A2 HUMAN
53	SRSF protein kinase 1	SRPK1 HUMAN
54	Thyrotropin-releasing hormone receptor	TRFR HUMAN
55	Tyrosine-protein kinase receptor UFO	UFO HUMAN
56	Vasopressin V2 receptor	V2R HUMAN
57	Alpha-2A adrenergic receptor	ADA2A HUMAN
58	Beta-2 adrenergic receptor	ADRB2 HUMAN
59	Type-1 angiotensin II receptor	AGTR1 HUMAN
60	Type-2 angiotensin II receptor	AGTR2 HUMAN
61	Aminopeptidase N	AMPN HUMAN
62	Androgen receptor	ANDR HUMAN
63	Sodium/potassium-transporting ATPase subunit beta-1	AT1B1 HUMAN
64	Sodium/potassium-transporting ATPase subunit beta-2	AT1B2 HUMAN
65	Caspase-1	CASP1 HUMAN
66	Cathepsin B	CATB HUMAN
67	Cyclin-dependent kinase 19	CDK19 HUMAN
68	Serine/threonine-protein kinase Chk1	CHK1 HUMAN
69	COUP transcription factor 2	COT2 HUMAN
70	Corticotropin-releasing factor receptor 1	CRFR1 HUMAN
71	Corticotropin-releasing factor receptor 2	CRFR2 HUMAN
72	Casein kinase II subunit alpha	CSK21 HUMAN
73	Casein kinase II subunit alpha′	CSK22 HUMAN
74	C-X-C chemokine receptor type 2	CXCR2 HUMAN
75	C-X-C chemokine receptor type 3	CXCR3 HUMAN
76	Death-associated protein kinase 3	DAPK3 HUMAN
77	Dipeptidyl peptidase 4	DPP4 HUMAN
78	Receptor tyrosine-protein kinase erbB-2	ERBB2 HUMAN
79	Receptor tyrosine-protein kinase erbB-3	ERBB3 HUMAN
80	Receptor tyrosine-protein kinase erbB-4	ERBB4 HUMAN
81	Focal adhesion kinase 1	FAK1 HUMAN
82	Protein-tyrosine kinase 2-beta	FAK2 HUMAN
83	Free fatty acid receptor 1	FFAR1 HUMAN
84	fMet-Leu-Phe receptor	FPR1 HUMAN
85	N-formyl peptide receptor 2	FPR2 HUMAN
86	Metabotropic glutamate receptor 5	GRM5 HUMAN
87	Inhibitor of nuclear factor kappa-B kinase subunit alpha	IKKA HUMAN
88	Inhibitor of nuclear factor kappa-B kinase subunit beta	IKKB HUMAN
89	Inhibitor of nuclear factor kappa-B kinase subunit epsilon	IKKE HUMAN
90	G protein-activated inward rectifier potassium channel 1	KCNJ3 HUMAN
91	G protein-activated inward rectifier potassium channel 4	KCNJ5 HUMAN
92	G protein-activated inward rectifier potassium channel 2	KCNJ6 HUMAN
93	KiSS-1 receptor	KISSR HUMAN
94	Ribosomal protein S6 kinase alpha-2	KS6A2 HUMAN
95	Tyrosine-protein kinase Lck	LCK HUMAN
96	Misshapen-like kinase 1	MINK1 HUMAN
97	Mitogen-activated protein kinase 8	MK08 HUMAN
98	Mitogen-activated protein kinase 9	MK09 HUMAN
99	Mitogen-activated protein kinase 10	MK10 HUMAN
100	Dual specificity mitogen-activated protein kinase kinase 2	MP2K2 HUMAN
101	Serine/threonine-protein kinase Nek1	NEK1 HUMAN
102	Serine/threonine-protein kinase Nek2	NEK2 HUMAN
103	Serine/threonine-protein kinase Nek4	NEK4 HUMAN
104	Mu-type opioid receptor	OPRM HUMAN

ID	Protein Name	UniProt Entry Name
105	Nociceptin receptor	OPRX HUMAN
106	Prostaglandin E2 receptor EP3 subtype	PE2R3 HUMAN
107	Prostaglandin E2 receptor EP4 subtype	PE2R4 HUMAN
108	Lysosomal protective protein	PPGB HUMAN
109	Nuclear receptor ROR-gamma	RORG HUMAN
110	Proto-oncogene tyrosine-protein kinase ROS	ROS1 HUMAN
111	Sphingosine 1-phosphate receptor 5	S1PR5 HUMAN
112	Solute carrier family 40 member 1	S40A1 HUMAN
113	Sodium-dependent dopamine transporter	SC6A3 HUMAN
114	Sodium-dependent serotonin transporter	SC6A4 HUMAN
115	Sodium- and chloride-dependent glycine transporter 2	SC6A5 HUMAN
116	Sodium channel protein type 1 subunit alpha	SCN1A HUMAN
117	Sodium channel protein type 2 subunit alpha	SCN2A HUMAN
118	Sodium/hydrogen exchanger 1	SL9A1 HUMAN
119	Sodium/hydrogen exchanger 3	SL9A3 HUMAN
120	Prothrombin	THRB HUMAN
121	Transient receptor potential cation channel subfamily V member 1	TRPV1 HUMAN
122	Trypsin-1	TRY1 HUMAN
123	Trypsin-2	TRY2 HUMAN
124	5-hydroxytryptamine receptor 1B	5HT1B HUMAN
125	5′-AMP-activated protein kinase catalytic subunit alpha-1	AAPK1 HUMAN
126	Angiotensin-converting enzyme	ACE HUMAN
127	Muscarinic acetylcholine receptor M1	ACM1 HUMAN
128	ALK tyrosine kinase receptor	ALK HUMAN
129	Sodium/potassium-transporting ATPase subunit alpha-3	AT1A3 HUMAN
130	Sodium/potassium-transporting ATPase subunit alpha-4	AT1A4 HUMAN
131	Cysteine protease ATG4B	ATG4B HUMAN
132	Breakpoint cluster region protein	BCR HUMAN
133	Bone morphogenetic protein 1	BMP1 HUMAN
134	Voltage-dependent calcium channel subunit alpha-2/delta-1	CA2D1 HUMAN
135	Dipeptidyl peptidase 1	CATC HUMAN
136	Complement factor D	CFAD HUMAN
137	Calcium release-activated calcium channel protein 1	CRCM1 HUMAN
138	Serine/threonine-protein kinase/endoribonuclease IRE1	ERN1 HUMAN
139	Estrogen receptor beta	ESR2 HUMAN
140	G protein-coupled receptor kinase 5	GRK5 HUMAN
141	Solute carrier family 2, facilitated glucose transporter member 1	GTR1 HUMAN
142	Hydroxycarboxylic acid receptor 2	HCAR2 HUMAN
143	cAMP-dependent protein kinase catalytic subunit alpha	KAPCA HUMAN
144	cAMP-dependent protein kinase catalytic subunit beta	KAPCB HUMAN
145	cAMP-dependent protein kinase catalytic subunit gamma	KAPCG HUMAN
146	Casein kinase I isoform epsilon	KC1E HUMAN
147	Casein kinase I isoform gamma-1	KC1G1 HUMAN
148	Casein kinase I isoform gamma-2	KC1G2 HUMAN
149	Casein kinase I isoform gamma-3	KC1G3 HUMAN
150	Kallikrein-1	KLK1 HUMAN
151	Leucyl-cystinyl aminopeptidase	LCAP HUMAN
152	LIM domain kinase 1	LIMK1 HUMAN
153	LIM domain kinase 2	LIMK2 HUMAN
154	Leukotriene B4 receptor 1	LT4R1 HUMAN
155	Mitogen-activated protein kinase 11	MK11 HUMAN
156	Mitogen-activated protein kinase 12	MK12 HUMAN
157	Mitogen-activated protein kinase 13	MK13 HUMAN

ID	Protein Name	UniProt Entry Name
158	Mitogen-activated protein kinase 14	MK14 HUMAN
159	Stromelysin-2	MMP10 HUMAN
160	Multidrug resistance-associated protein 1	MRP1 HUMAN
161	Serine/threonine-protein kinase mTOR	MTOR HUMAN
162	Glutamate receptor ionotropic, NMDA 1	NMDZ1 HUMAN
163	Neuropeptide Y receptor type 2	NPY2R HUMAN
164	Neuropeptide Y receptor type 5	NPY5R HUMAN
165	Photoreceptor-specific nuclear receptor	NR2E3 HUMAN
166	P2Y purinoceptor 2	P2RY2 HUMAN
167	Serine/threonine-protein kinase PAK 1	PAK1 HUMAN
168	Serine/threonine-protein kinase PAK 4	PAK4 HUMAN
169	Serine/threonine-protein kinase PLK1	PLK1 HUMAN
170	Prolyl endopeptidase	PPCE HUMAN
171	26S proteasome non-ATPase regulatory subunit 14	PSDE HUMAN
172	Presenilin-2	PSN2 HUMAN
173	Parathyroid hormone/parathyroid hormone-related peptide receptor	PTH1R HUMAN
174	Protein-tyrosine kinase 6	PTK6 HUMAN
175	Retinoic acid receptor alpha	RARA HUMAN
176	Retinoic acid receptor beta	RARB HUMAN
177	Rho-associated protein kinase 1	ROCK1 HUMAN
178	Rho-associated protein kinase 2	ROCK2 HUMAN
179	High affinity choline transporter 1	SC5A7 HUMAN
180	Solute carrier organic anion transporter family member 1B1	SO1B1 HUMAN
181	Thyroid hormone receptor alpha	THA HUMAN
182	Transmembrane protease serine 6	TMPS6 HUMAN
183	Urokinase-type plasminogen activator	UROK HUMAN
184	Vasopressin V1b receptor	V1BR HUMAN
185	Tyrosine-protein kinase ZAP-70	ZAP70 HUMAN
186	5-hydroxytryptamine receptor 7	5HT7R HUMAN
187	Neuronal acetylcholine receptor subunit beta-4	ACHB4 HUMAN
188	Acetylcholine receptor subunit beta	ACHB HUMAN
189	Activin receptor type-1	ACVR1 HUMAN
190	Sodium/potassium-transporting ATPase subunit beta-3	AT1B3 HUMAN
191	Voltage-dependent N-type calcium channel subunit alpha-1B	CAC1B HUMAN
192	Voltage-dependent L-type calcium channel subunit alpha-1C	CAC1C HUMAN
193	Voltage-dependent L-type calcium channel subunit alpha-1D	CAC1D HUMAN
194	Extracellular calcium-sensing receptor	CASR HUMAN
195	Carboxypeptidase B2	CBPB2 HUMAN
196	C-C chemokine receptor type 9	CCR9 HUMAN
197	Cyclin-dependent kinase 7	CDK7 HUMAN
198	Cyclin-dependent kinase 8	CDK8 HUMAN
199	Chemokine-like receptor 1	CML1 HUMAN
200	Cannabinoid receptor 2	CNR2 HUMAN
201	Dipeptidyl peptidase 2	DPP2 HUMAN
202	Endothelin-1 receptor	EDNRA HUMAN
203	Endothelin receptor type B	EDNRB HUMAN
204	CAAX prenyl protease 2	FACE2 HUMAN
205	Gastrin/cholecystokinin type B receptor	GASR HUMAN
206	Gamma-aminobutyric acid receptor subunit theta	GBRT HUMAN
207	Glucagon-like peptide 1 receptor	GLP1R HUMAN
208	Calcium/calmodulin-dependent protein kinase type II subunit beta	KCC2B HUMAN
209	ATP-sensitive inward rectifier potassium channel 1	KCNJ1 HUMAN
210	Potassium voltage-gated channel subfamily KQT member 2	KCNQ2 HUMAN

ID	Protein Name	UniProt Entry Name
211	Potassium voltage-gated channel subfamily KQT member 3	KCNQ3 HUMAN
212	Protein kinase C theta type	KPCT HUMAN
213	Protein kinase C zeta type	KPCZ HUMAN
214	Mitogen-activated protein kinase kinase kinase 20	M3K20 HUMAN
215	Dual specificity mitogen-activated protein kinase kinase 1	MP2K1 HUMAN
216	Nuclear receptor subfamily 5 group A member 2	NR5A2 HUMAN
217	Proteinase-activated receptor 4	PAR4 HUMAN
218	PAS domain-containing serine/threonine-protein kinase	PASK HUMAN
219	Peroxisome proliferator-activated receptor alpha	PPARA HUMAN
220	Peroxisome proliferator-activated receptor delta	PPARD HUMAN
221	Peroxisome proliferator-activated receptor gamma	PPARG HUMAN
222	Renin	RENI HUMAN
223	Sodium channel protein type 9 subunit alpha	SCN9A HUMAN
224	Suppressor of tumorigenicity 14 protein	ST14 HUMAN
225	Serine/threonine-protein kinase 17A	ST17A HUMAN
226	Serine/threonine-protein kinase 3	STK3 HUMAN
227	Urotensin-2 receptor	UR2R HUMAN
228	Tyrosine-protein kinase Yes	YES HUMAN
229	Neuronal acetylcholine receptor subunit alpha-3	ACHA3 HUMAN
230	Neuronal acetylcholine receptor subunit alpha-4	ACHA4 HUMAN
231	Disintegrin and metalloproteinase domain-containing protein 17	ADA17 HUMAN
232	Alpha-1A adrenergic receptor	ADA1A HUMAN
233	Potassium-transporting ATPase alpha chain 1	ATP4A HUMAN
234	Potassium-transporting ATPase subunit beta	ATP4B HUMAN
235	Tyrosine-protein kinase Blk	BLK HUMAN
236	Caspase-6	CASP6 HUMAN
237	Caspase-7	CASP7 HUMAN
238	C-C chemokine receptor type 1	CCR1 HUMAN
239	C-C chemokine receptor type 2	CCR2 HUMAN
240	C-C chemokine receptor type 3	CCR3 HUMAN
241	Cyclin-dependent kinase 4	CDK4 HUMAN
242	Cyclin-dependent-like kinase 5	CDK5 HUMAN
243	Cyclin-dependent kinase 6	CDK6 HUMAN
244	Dual specificity protein kinase CLK2	CLK2 HUMAN
245	Dual specificity protein kinase CLK4	CLK4 HUMAN
246	Chymase	CMA1 HUMAN
247	Cannabinoid receptor 1	CNR1 HUMAN
248	Endoplasmic reticulum aminopeptidase 1	ERAP1 HUMAN
249	Endoplasmic reticulum aminopeptidase 2	ERAP2 HUMAN
250	Coagulation factor VII	FA7 HUMAN
251	Free fatty acid receptor 4	FFAR4 HUMAN
252	Fibroblast growth factor receptor 1	FGFR1 HUMAN
253	Receptor-type tyrosine-protein kinase FLT3	FLT3 HUMAN
254	Gamma-aminobutyric acid receptor subunit alpha-2	GBRA2 HUMAN
255	Gamma-aminobutyric acid receptor subunit alpha-3	GBRA3 HUMAN
256	Gamma-aminobutyric acid receptor subunit alpha-4	GBRA4 HUMAN
257	Gamma-aminobutyric acid receptor subunit alpha-5	GBRA5 HUMAN
258	Gamma-aminobutyric acid receptor subunit alpha-6	GBRA6 HUMAN
259	Growth hormone secretagogue receptor type 1	GHSR HUMAN
260	G-protein coupled receptor 84	GPR84 HUMAN
261	Glutamate receptor 1	GRIA1 HUMAN
262	Glutamate receptor 2	GRIA2 HUMAN
263	Glutamate receptor 3	GRIA3 HUMAN

ID	Protein Name	UniProt Entry Name
264	Glutamate receptor 4	GRIA4 HUMAN
265	Glycogen synthase kinase-3 alpha	GSK3A HUMAN
266	Glycogen synthase kinase-3 beta	GSK3B HUMAN
267	Mitogen-activated protein kinase kinase kinase kinase 4	M4K4 HUMAN
268	Mitogen-activated protein kinase kinase kinase kinase 5	M4K5 HUMAN
269	Methionine aminopeptidase 2	MAP2 HUMAN
270	ATP-dependent translocase ABCB1	MDR1 HUMAN
271	Neutrophil collagenase	MMP8 HUMAN
272	Matrix metalloproteinase-9	MMP9 HUMAN
273	Orexin receptor type 2	OX2R HUMAN
274	Oxytocin receptor	OXYR HUMAN
275	P2X purinoceptor 2	P2RX2 HUMAN
276	Prostaglandin D2 receptor 2	PD2R2 HUMAN
277	Prostaglandin D2 receptor	PD2R HUMAN
278	Prostacyclin receptor	PI2R HUMAN
279	Platelet-activating factor receptor	PTAFR HUMAN
280	Relaxin receptor 1	RXFP1 HUMAN
281	Sphingosine 1-phosphate receptor 1	S1PR1 HUMAN
282	Sphingosine 1-phosphate receptor 2	S1PR2 HUMAN
283	Sphingosine 1-phosphate receptor 3	S1PR3 HUMAN
284	Sphingosine 1-phosphate receptor 4	S1PR4 HUMAN
285	Sodium/glucose cotransporter 1	SC5A1 HUMAN
286	Sodium/glucose cotransporter 2	SC5A2 HUMAN
287	Taste receptor type 2 member 8	TA2R8 HUMAN
288	Taste receptor type 1 member 1	TS1R1 HUMAN
289	Taste receptor type 1 member 3	TS1R3 HUMAN
290	Tyrosine-protein kinase receptor TYRO3	TYRO3 HUMAN
291	5-hydroxytryptamine receptor 1A	5HT1A HUMAN
292	Adenosine receptor A3	AA3R HUMAN
293	Tyrosine-protein kinase ABL1	ABL1 HUMAN
294	Tyrosine-protein kinase ABL2	ABL2 HUMAN
295	Angiotensin-converting enzyme 2	ACE2 HUMAN
296	Acetylcholine receptor subunit delta	ACHD HUMAN
297	Acetylcholine receptor subunit gamma	ACHG HUMAN
298	Sodium/potassium-transporting ATPase subunit alpha-1	AT1A1 HUMAN
299	Sodium/potassium-transporting ATPase subunit alpha-2	AT1A2 HUMAN
300	Complement C1s subcomponent	C1S HUMAN
301	Cathepsin L1	CATL1 HUMAN
302	Cathepsin L2	CATL2 HUMAN
303	Cyclin-dependent kinase 2	CDK2 HUMAN
304	D(2) dopamine receptor	DRD2 HUMAN
305	D(3) dopamine receptor	DRD3 HUMAN
306	D(4) dopamine receptor	DRD4 HUMAN
307	D(1B) dopamine receptor	DRD5 HUMAN
308	Dual specificity tyrosine-phosphorylation-regulated kinase 2	DYRK2 HUMAN
309	Dual specificity tyrosine-phosphorylation-regulated kinase 3	DYRK3 HUMAN
310	Coagulation factor X	FA10 HUMAN
311	Gamma-aminobutyric acid receptor subunit gamma-3	GBRG3 HUMAN
312	Gamma-aminobutyric acid receptor subunit pi	GBRP HUMAN
313	Gonadotropin-releasing hormone receptor	GNRHR HUMAN
314	Glutamate receptor ionotropic, kainate 2	GRIK2 HUMAN
315	Interleukin-1 receptor-associated kinase 4	IRAK4 HUMAN
316	Calcium/calmodulin-dependent protein kinase type II subunit delta	KCC2D HUMAN

ID	Protein Name	UniProt Entry Name
317	Calcium/calmodulin-dependent protein kinase type II subunit gamma	KCC2G HUMAN
318	ATP-sensitive inward rectifier potassium channel 8	KCNJ8 HUMAN
319	Potassium channel subfamily K member 3	KCNK3 HUMAN
320	Protein kinase C delta type	KPCD HUMAN
321	Protein kinase C epsilon type	KPCE HUMAN
322	Protein kinase C gamma type	KPCG HUMAN
323	Protein kinase C iota type	KPCI HUMAN
324	Protein kinase C eta type	KPCL HUMAN
325	Ribosomal protein S6 kinase alpha-3	KS6A3 HUMAN
326	Ribosomal protein S6 kinase alpha-5	KS6A5 HUMAN
327	Ribosomal protein S6 kinase beta-1	KS6B1 HUMAN
328	Mitogen-activated protein kinase kinase kinase kinase 2	M4K2 HUMAN
329	Methionine aminopeptidase 1	MAP11 HUMAN
330	Melanin-concentrating hormone receptor 1	MCHR1 HUMAN
331	Maternal embryonic leucine zipper kinase	MELK HUMAN
332	Melanocyte-stimulating hormone receptor	MSHR HUMAN
333	Melatonin receptor type 1B	MTR1B HUMAN
334	Nicastrin	NICA HUMAN
335	Proteinase-activated receptor 1	PAR1 HUMAN
336	Lysosomal Pro-X carboxypeptidase	PCP HUMAN
337	Proprotein convertase subtilisin/kexin type 6	PCSK6 HUMAN
338	Platelet-derived growth factor receptor beta	PGFRB HUMAN
339	Serine/threonine-protein kinase N2	PKN2 HUMAN
340	Serine/threonine-protein kinase PLK4	PLK4 HUMAN
341	Vitamin K-dependent protein C	PROC HUMAN
342	Receptor-interacting serine/threonine-protein kinase 1	RIPK1 HUMAN
343	Somatostatin receptor type 3	SSR3 HUMAN
344	Somatostatin receptor type 4	SSR4 HUMAN
345	Somatostatin receptor type 5	SSR5 HUMAN
346	Thromboxane A2 receptor	TA2R HUMAN
347	Serine/threonine-protein kinase TAO1	TAOK1 HUMAN
348	Serine/threonine-protein kinase TNNI3K	TNI3K HUMAN
349	TRAF2 and NCK-interacting protein kinase	TNIK HUMAN
350	Ubiquitin carboxyl-terminal hydrolase 1	UBP1 HUMAN
351	Vesicular acetylcholine transporter	VACHT HUMAN
352	ATP-binding cassette sub-family G member 2	ABCG2 HUMAN
353	Acetyl-coenzyme A transporter 1	ACATN HUMAN
354	Muscarinic acetylcholine receptor M2	ACM2 HUMAN
355	Muscarinic acetylcholine receptor M3	ACM3 HUMAN
356	Alpha-2B adrenergic receptor	ADA2B HUMAN
357	Alpha-2C adrenergic receptor	ADA2C HUMAN
358	Beta-3 adrenergic receptor	ADRB3 HUMAN
359	Sodium/potassium-transporting ATPase subunit gamma	ATNG HUMAN
360	Bromodomain-containing protein 4	BRD4 HUMAN
361	Caspase-8	CASP8 HUMAN
362	C-C chemokine receptor type 4	CCR4 HUMAN
363	Cyclin-dependent kinase 1	CDK1 HUMAN
364	Dipeptidyl peptidase 8	DPP8 HUMAN
365	Dipeptidyl peptidase 9	DPP9 HUMAN
366	Tyrosine-protein kinase FRK	FRK HUMAN
367	Tyrosine-protein kinase Fyn	FYN HUMAN
368	Glucose-dependent insulinotropic receptor	GP119 HUMAN
369	G-protein coupled receptor 35	GPR35 HUMAN

ID	Protein Name	UniProt Entry Name
370	Metabotropic glutamate receptor 1	GRM1 HUMAN
371	Metabotropic glutamate receptor 2	GRM2 HUMAN
372	Metabotropic glutamate receptor 4	GRM4 HUMAN
373	Hepatocyte nuclear factor 4-alpha	HNF4A HUMAN
374	Tyrosine-protein kinase ITK/TSK	ITK HUMAN
375	ATP-sensitive inward rectifier potassium channel 11	KCJ11 HUMAN
376	Potassium voltage-gated channel subfamily H member 2	KCNH2 HUMAN
377	Protein kinase C alpha type	KPCA HUMAN
378	Protein kinase C beta type	KPCB HUMAN
379	Serine/threonine-protein kinase D1	KPCD1 HUMAN
380	Serine/threonine-protein kinase D2	KPCD2 HUMAN
381	Serine/threonine-protein kinase D3	KPCD3 HUMAN
382	Leukotriene B4 receptor 2	LT4R2 HUMAN
383	Mineralocorticoid receptor	MCR HUMAN
384	Tyrosine-protein kinase Mer	MERTK HUMAN
385	Sodium/calcium exchanger 1	NAC1 HUMAN
386	Glutamate receptor ionotropic, NMDA 2A	NMDE1 HUMAN
387	Glutamate receptor ionotropic, NMDA 2B	NMDE2 HUMAN
388	Glutamate receptor ionotropic, NMDA 2C	NMDE3 HUMAN
389	Glutamate receptor ionotropic, NMDA 2D	NMDE4 HUMAN
390	Oxysterols receptor LXR-beta	NR1H2 HUMAN
391	Oxysterols receptor LXR-alpha	NR1H3 HUMAN
392	Bile acid receptor	NR1H4 HUMAN
393	P2X purinoceptor 3	P2RX3 HUMAN
394	P2X purinoceptor 7	P2RX7 HUMAN
395	P2Y purinoceptor 1	P2RY1 HUMAN
396	3-phosphoinositide-dependent protein kinase 1	PDPK1 HUMAN
397	Phosphorylase b kinase gamma catalytic chain, liver/testis isoform	PHKG2 HUMAN
398	Sodium channel protein type 3 subunit alpha	SCN3A HUMAN
399	Sodium channel protein type 5 subunit alpha	SCN5A HUMAN
400	Sentrin-specific protease 6	SENP6 HUMAN
401	Sentrin-specific protease 7	SENP7 HUMAN
402	Sentrin-specific protease 8	SENP8 HUMAN
403	Serine/threonine-protein kinase Sgk2	SGK2 HUMAN
404	Solute carrier organic anion transporter family member 1B3	SO1B3 HUMAN
405	Steroidogenic factor 1	STF1 HUMAN
406	Trace amine-associated receptor 1	TAAR1 HUMAN
407	Vascular endothelial growth factor receptor 1	VGFR1 HUMAN
408	5-hydroxytryptamine receptor 2A	5HT2A HUMAN
409	5-hydroxytryptamine receptor 2B	5HT2B HUMAN
410	5-hydroxytryptamine receptor 2C	5HT2C HUMAN
411	5′-AMP-activated protein kinase catalytic subunit alpha-2	AAPK2 HUMAN
412	Bile salt export pump	ABCBB HUMAN
413	Neuronal acetylcholine receptor subunit alpha-7	ACHA7 HUMAN
414	Atypical chemokine receptor 3	ACKR3 HUMAN
415	Disintegrin and metalloproteinase domain-containing protein 10	ADA10 HUMAN
416	Bombesin receptor subtype-3	BRS3 HUMAN
417	Serine/threonine-protein kinase BRSK1	BRSK1 HUMAN
418	Tyrosine-protein kinase BTK	BTK HUMAN
419	Calcitonin gene-related peptide type 1 receptor	CALRL HUMAN
420	Caspase-3	CASP3 HUMAN
421	Cathepsin D	CATD HUMAN
422	Cholecystokinin receptor type A	CCKAR HUMAN

ID	Protein Name	UniProt Entry Name
423	Dual specificity protein kinase CLK1	CLK1 HUMAN
424	Cysteinyl leukotriene receptor 1	CLTR1 HUMAN
425	Cysteinyl leukotriene receptor 2	CLTR2 HUMAN
426	C-X-C chemokine receptor type 4	CXCR4 HUMAN
427	Enteropeptidase	ENTK HUMAN
428	Steroid hormone receptor ERR1	ERR1 HUMAN
429	Furin	FURIN HUMAN
430	Gamma-aminobutyric acid receptor subunit delta	GBRD HUMAN
431	Gamma-aminobutyric acid receptor subunit epsilon	GBRE HUMAN
432	Gamma-aminobutyric acid receptor subunit gamma-1	GBRG1 HUMAN
433	Gamma-aminobutyric acid receptor subunit gamma-2	GBRG2 HUMAN
434	Glucocorticoid receptor	GCR HUMAN
435	G-protein coupled bile acid receptor 1	GPBAR HUMAN
436	Tyrosine-protein kinase HCK	HCK HUMAN
437	Interleukin-1 receptor-associated kinase 1	IRAK1 HUMAN
438	Hepatocyte growth factor receptor	MET HUMAN
439	Neprilysin	NEP HUMAN
440	Neuromedin-K receptor	NK3R HUMAN
441	Neuropeptides B/W receptor type 1	NPBW1 HUMAN
442	NPC intracellular cholesterol transporter 1	NPC1 HUMAN
443	Neuropeptide Y receptor type 1	NPY1R HUMAN
444	Nuclear receptor subfamily 1 group I member 2	NR1I2 HUMAN
445	Neurotensin receptor type 1	NTR1 HUMAN
446	Serine/threonine-protein kinase pim-1	PIM1 HUMAN
447	Serine/threonine-protein kinase pim-2	PIM2 HUMAN
448	Serine/threonine-protein kinase pim-3	PIM3 HUMAN
449	RAF proto-oncogene serine/threonine-protein kinase	RAF1 HUMAN
450	Retinoic acid receptor RXR-gamma	RXRG HUMAN
451	Solute carrier family 22 member 12	S22AC HUMAN
452	Equilibrative nucleoside transporter 1	S29A1 HUMAN
453	Vascular endothelial growth factor receptor 2	VGFR2 HUMAN
454	Vascular endothelial growth factor receptor 3	VGFR3 HUMAN
455	Wee1-like protein kinase	WEE1 HUMAN
456	5-hydroxytryptamine receptor 3A	5HT3A HUMAN
457	5-hydroxytryptamine receptor 3B	5HT3B HUMAN
458	5-hydroxytryptamine receptor 3C	5HT3C HUMAN
459	5-hydroxytryptamine receptor 3D	5HT3D HUMAN
460	5-hydroxytryptamine receptor 3E	5HT3E HUMAN
461	5-hydroxytryptamine receptor 4	5HT4R HUMAN
462	5-hydroxytryptamine receptor 5A	5HT5A HUMAN
463	5-hydroxytryptamine receptor 6	5HT6R HUMAN
464	ATP-binding cassette sub-family C member 8	ABCC8 HUMAN
465	ATP-binding cassette sub-family C member 9	ABCC9 HUMAN
466	Muscarinic acetylcholine receptor M4	ACM4 HUMAN
467	Muscarinic acetylcholine receptor M5	ACM5 HUMAN
468	Beta-1 adrenergic receptor	ADRB1 HUMAN
469	RAC-alpha serine/threonine-protein kinase	AKT1 HUMAN
470	RAC-beta serine/threonine-protein kinase	AKT2 HUMAN
471	RAC-gamma serine/threonine-protein kinase	AKT3 HUMAN
472	Serine-protein kinase ATM	ATM HUMAN
473	C5a anaphylatoxin chemotactic receptor 1	C5AR1 HUMAN
474	Voltage-dependent L-type calcium channel subunit alpha-1F	CAC1F HUMAN
475	Voltage-dependent T-type calcium channel subunit alpha-1G	CAC1G HUMAN

ID	Protein Name	UniProt Entry Name
476	Voltage-dependent T-type calcium channel subunit alpha-1H	CAC1H HUMAN
477	Voltage-dependent T-type calcium channel subunit alpha-1I	CAC1I HUMAN
478	Voltage-dependent L-type calcium channel subunit alpha-1S	CAC1S HUMAN
479	Calpain-1 catalytic subunit	CAN1 HUMAN
480	Calpain-2 catalytic subunit	CAN2 HUMAN
481	Cathepsin K	CATK HUMAN
482	Cystic fibrosis transmembrane conductance regulator	CFTR HUMAN
483	Serine/threonine-protein kinase Chk2	CHK2 HUMAN
484	Epithelial discoidin domain-containing receptor 1	DDR1 HUMAN
485	D(1A) dopamine receptor	DRD1 HUMAN
486	Dual specificity tyrosine-phosphorylation-regulated kinase 1A	DYR1A HUMAN
487	Dual specificity tyrosine-phosphorylation-regulated kinase 1B	DYR1B HUMAN
488	Endothelin-converting enzyme 1	ECE1 HUMAN
489	Neutrophil elastase	ELNE HUMAN
490	Coagulation factor IX	FA9 HUMAN
491	Glutamate carboxypeptidase 2	FOLH1 HUMAN
492	Homeodomain-interacting protein kinase 2	HIPK2 HUMAN
493	Homeodomain-interacting protein kinase 4	HIPK4 HUMAN
494	Histamine H1 receptor	HRH1 HUMAN
495	Histamine H2 receptor	HRH2 HUMAN
496	Histamine H3 receptor	HRH3 HUMAN
497	Histamine H4 receptor	HRH4 HUMAN
498	Epoxide hydrolase 1	HYEP HUMAN
499	Tyrosine-protein kinase JAK2	JAK2 HUMAN
500	Tyrosine-protein kinase JAK1	JAK1 HUMAN
501	Tyrosine-protein kinase JAK3	JAK3 HUMAN
502	Casein kinase I isoform alpha	KC1A HUMAN
503	Casein kinase I isoform delta	KC1D HUMAN
504	Leucine-rich repeat serine/threonine-protein kinase 2	LRRK2 HUMAN
505	Mitogen-activated protein kinase 3	MK03 HUMAN
506	Mitogen-activated protein kinase 1	MK01 HUMAN
507	Interstitial collagenase	MMP1 HUMAN
508	Motilin receptor	MTLR HUMAN
509	Substance-P receptor	NK1R HUMAN
510	Substance-K receptor	NK2R HUMAN
511	Neuropeptide S receptor	NPSR1 HUMAN
512	Ileal sodium/bile acid cotransporter	NTCP2 HUMAN
513	High affinity nerve growth factor receptor	NTRK1 HUMAN
514	BDNF/NT-3 growth factors receptor	NTRK2 HUMAN
515	NT-3 growth factor receptor	NTRK3 HUMAN
516	P2Y purinoceptor 12	P2Y12 HUMAN
517	[Pyruvate dehydrogenase (acetyl-transferring)] kinase isozyme 1, mitochondrial	PDK1 HUMAN
518	[Pyruvate dehydrogenase (acetyl-transferring)] kinase isozyme 2, mitochondrial	PDK2 HUMAN
519	[Pyruvate dehydrogenase (acetyl-transferring)] kinase isozyme 3, mitochondrial	PDK3 HUMAN
520	[Pyruvate dehydrogenase (acetyl-transferring)] kinase isozyme 4, mitochondrial	PDK4 HUMAN
521	Prostaglandin E2 receptor EP1 subtype	PE2R1 HUMAN
522	Prostaglandin E2 receptor EP2 subtype	PE2R2 HUMAN
523	Perforin-1	PERF HUMAN
524	Serine/threonine-protein kinase PLK3	PLK3 HUMAN

ID	Protein Name	UniProt Entry Name
525	Sodium- and chloride-dependent glycine transporter 1	SC6A9 HUMAN
526	Sodium channel protein type 10 subunit alpha	SCNAA HUMAN
527	Sentrin-specific protease 1	SENP1 HUMAN
528	Prolyl endopeptidase FAP	SEPR HUMAN
529	STE20-like serine/threonine-protein kinase	SLK HUMAN
530	Smoothened homolog	SMO HUMAN
531	Somatostatin receptor type 1	SSR1 HUMAN
532	Somatostatin receptor type 2	SSR2 HUMAN
533	Serine/threonine-protein kinase TBK1	TBK1 HUMAN
534	TGF-beta receptor type-1	TGFR1 HUMAN
535	Lymphokine-activated killer T-cell-originated protein kinase	TOPK HUMAN
536	Transient receptor potential cation channel subfamily V member 3	TRPV3 HUMAN
537	Transient receptor potential cation channel subfamily V member 4	TRPV4 HUMAN
538	Trypsin-3	TRY3 HUMAN
539	Non-receptor tyrosine-protein kinase TYK2	TYK2 HUMAN
540	5-hydroxytryptamine receptor 1D	5HT1D HUMAN
541	Acetylcholine receptor subunit alpha	ACHA HUMAN
542	Neuronal acetylcholine receptor subunit beta-2	ACHB2 HUMAN
543	Alpha-1B adrenergic receptor	ADA1B HUMAN
544	Alpha-1D adrenergic receptor	ADA1D HUMAN
545	Beta-adrenergic receptor kinase 1	ARBK1 HUMAN
546	A disintegrin and metalloproteinase with thrombospondin motifs 4	ATS4 HUMAN
547	A disintegrin and metalloproteinase with thrombospondin motifs 5	ATS5 HUMAN
548	Cathepsin G	CATG HUMAN
549	Cell division cycle 7-related protein kinase	CDC7 HUMAN
550	C-X-C chemokine receptor type 1	CXCR1 HUMAN
551	Epidermal growth factor receptor	EGFR HUMAN
552	Ephrin type-B receptor 4	EPHB4 HUMAN
553	Estrogen receptor	ESR1 HUMAN
554	Coagulation factor XI	FA11 HUMAN
555	Coagulation factor XII	FA12 HUMAN
556	Fibroblast growth factor receptor 2	FGFR2 HUMAN
557	Fibroblast growth factor receptor 3	FGFR3 HUMAN
558	Fibroblast growth factor receptor 4	FGFR4 HUMAN
559	Gamma-aminobutyric acid receptor subunit beta-1	GBRB1 HUMAN
560	Gamma-aminobutyric acid receptor subunit beta-2	GBRB2 HUMAN
561	Gamma-aminobutyric acid receptor subunit beta-3	GBRB3 HUMAN
562	Glutamate receptor ionotropic, kainate 1	GRIK1 HUMAN
563	Integrin-linked protein kinase	ILK HUMAN
564	Insulin receptor	INSR HUMAN
565	Kallikrein-7	KLK7 HUMAN
566	Plasma kallikrein	KLKB1 HUMAN
567	Leukotriene A-4 hydrolase	LKHA4 HUMAN
568	Tyrosine-protein kinase Lyn	LYN HUMAN
569	Mitogen-activated protein kinase kinase kinase 5	M3K5 HUMAN
570	Mitogen-activated protein kinase kinase kinase 7	M3K7 HUMAN
571	Mitogen-activated protein kinase kinase kinase 8	M3K8 HUMAN
572	Serine/threonine-protein kinase MARK2	MARK2 HUMAN
573	MAP/microtubule affinity-regulating kinase 3	MARK3 HUMAN
574	Macrophage metalloelastase	MMP12 HUMAN
575	Collagenase 3	MMP13 HUMAN
576	Matrix metalloproteinase-14	MMP14 HUMAN
577	Serine/threonine-protein kinase MRCK alpha	MRCKA HUMAN

ID	Protein Name	UniProt Entry Name
578	Glutamate receptor ionotropic, NMDA 3A	NMD3A HUMAN
579	Glutamate receptor ionotropic, NMDA 3B	NMD3B HUMAN
580	Nuclear receptor subfamily 4 group A member 1	NR4A1 HUMAN
581	Delta-type opioid receptor	OPRD HUMAN
582	Kappa-type opioid receptor	OPRK HUMAN
583	Orexin receptor type 1	OX1R HUMAN
584	Plasminogen	PLMN HUMAN
585	Proto-oncogene tyrosine-protein kinase receptor Ret	RET HUMAN
586	Proto-oncogene tyrosine-protein kinase Src	SRC HUMAN
587	Thyroid hormone receptor beta	THB HUMAN
588	Angiopoietin-1 receptor	TIE2 HUMAN
589	Tissue-type plasminogen activator	TPA HUMAN
590	Transient receptor potential cation channel subfamily A member 1	TRPA1 HUMAN
591	Transient receptor potential cation channel subfamily M member 8	TRPM8 HUMAN
592	Tryptase alpha/beta-1	TRYB1 HUMAN
593	Tryptase delta	TRYD HUMAN
594	Tryptase gamma	TRYG1 HUMAN
595	Thyrotropin receptor	TSHR HUMAN
596	Dual specificity protein kinase TTK	TTK HUMAN
597	Ubiquitin carboxyl-terminal hydrolase 2	UBP2 HUMAN
598	Vasopressin V1a receptor	V1AR HUMAN
599	Vitamin D3 receptor	VDR HUMAN

Table S2. List of reference compounds used in CGBSP.

ChEMBL ID	Structure	IC_{50} (nM)
CHEMBL437612	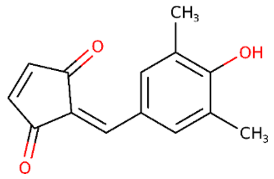	440
CHEMBL1093057	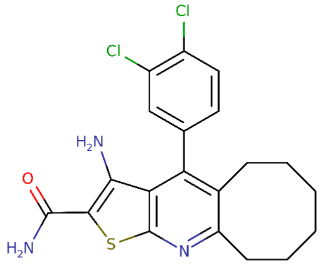	990
CHEMBL1091317	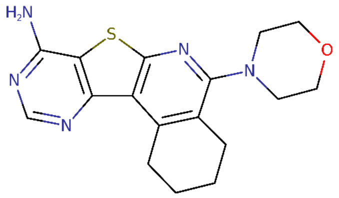	220

**ChEMBL ID**	**Structure**	**IC_{50} (nM)**
CHEMBL1089016	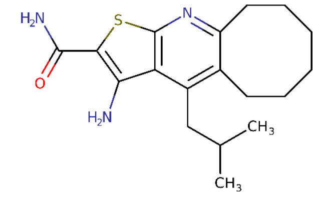	860
CHEMBL1090045	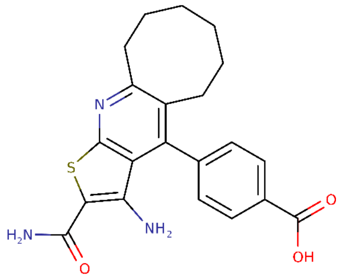	860
CHEMBL1094038	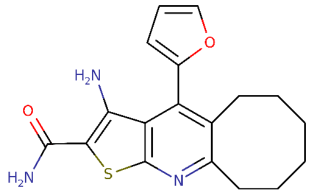	640
CHEMBL1091829	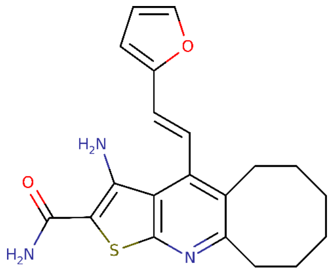	320
CHEMBL1091830	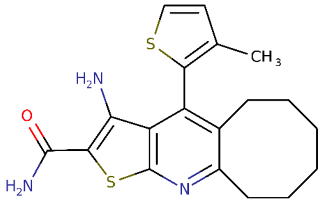	460
CHEMBL1089330	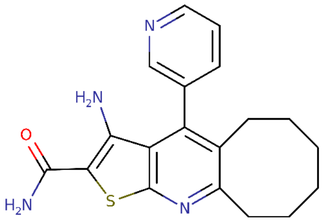	940
CHEMBL1092378	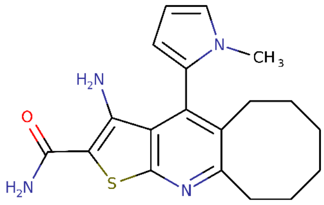	990

**ChEMBL ID**	**Structure**	**IC_{50} (nM)**
CHEMBL1094026	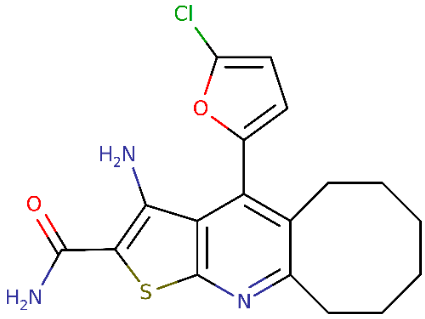	280
CHEMBL1089019	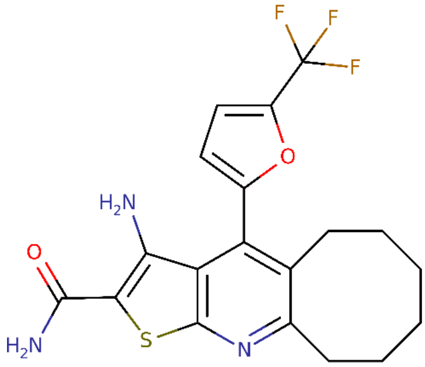	300
CHEMBL1091074	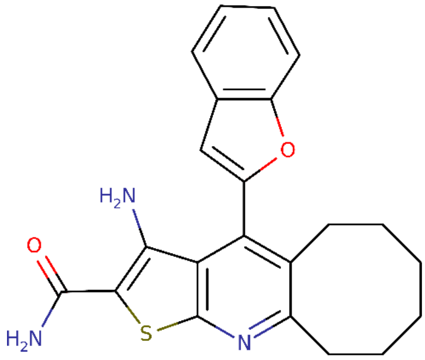	330
CHEMBL1091075	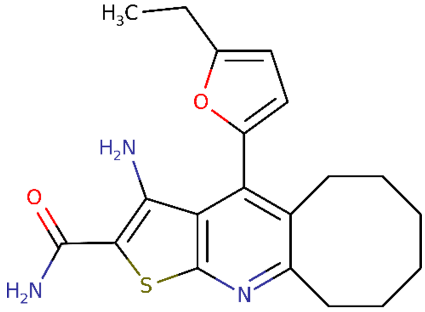	660

ChEMBL ID	Structure	IC_{50} (nM)
CHEMBL1091459	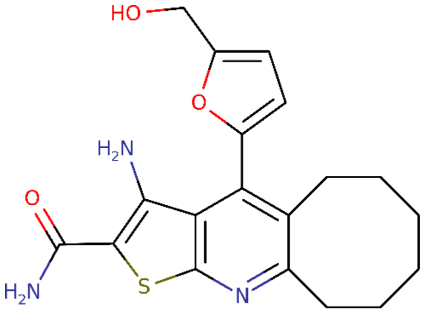	800
CHEMBL1092820	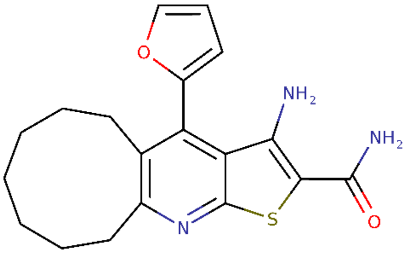	170
CHEMBL1091450	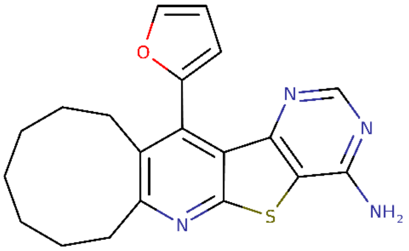	350
CHEMBL1094018	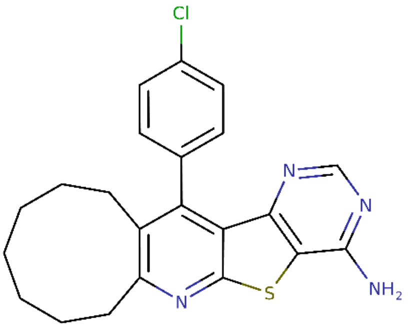	110
CHEMBL3327225	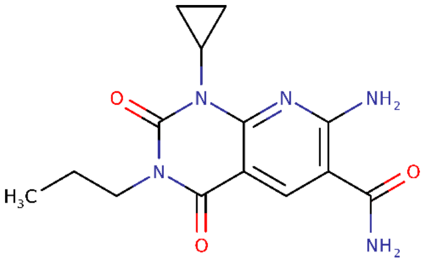	930

ChEMBL ID	Structure	IC_{50} (nM)
CHEMBL1977874	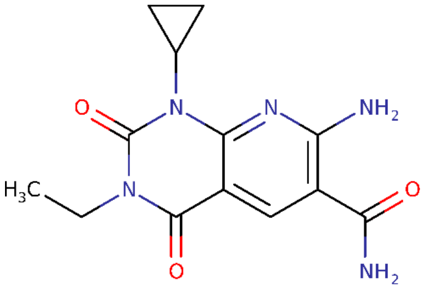	280

Table S3. List of compounds tested for *in vitro* inhibitory activity.

ID	Structure	Enamine ID	Reference[1]	Similarity[2]	% Inh.
compound1	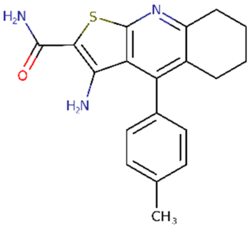	Z90664459	CHEMBL1089330	0.729412	14.5
compound2	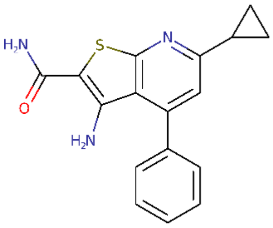	Z57147099	CHEMBL1089330	0.415842	74.1
compound3	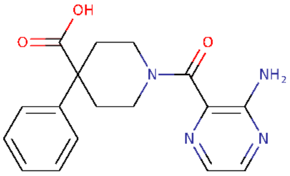	Z160320077 1	CHEMBL1090045	0.195122	2
compound4	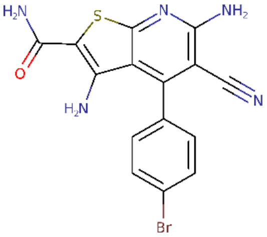	Z57076017	CHEMBL1090045	0.44898	29.4
compound5	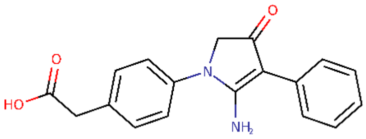	Z56823152	CHEMBL1090045	0.2	-5.2

ID	Structure	Enamine ID	Reference[1]	Similarity[2]	% Inh.
compound6	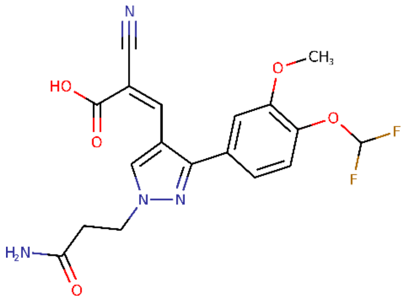	Z56347366	CHEMBL1090045	0.179104	-0.2
compound7	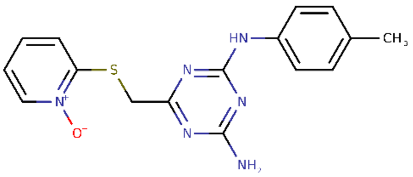	Z51103017	CHEMBL1090045	0.161538	-5.7
compound8	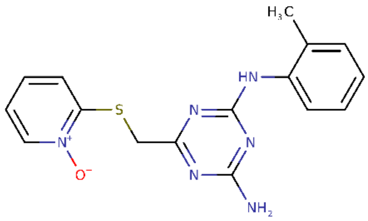	Z18546119	CHEMBL1090045	0.162791	-0.2
compound9	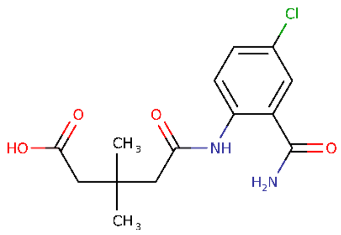	Z841115344	CHEMBL1090045	0.1875	2
compound10	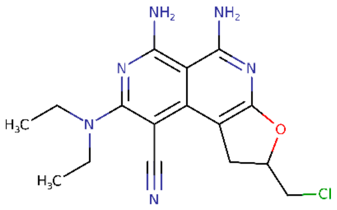	Z56172877	CHEMBL3327225	0.168224	5.7
compound11	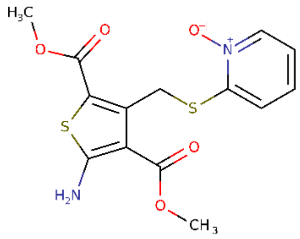	Z18545629	CHEMBL1090045	0.173554	3.9
compound12	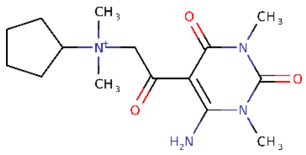	Z48843107	CHEMBL1090045	0.266667	-2.1

ID	Structure	Enamine ID	Reference[1]	Similarity[2]	% Inh.
compound13	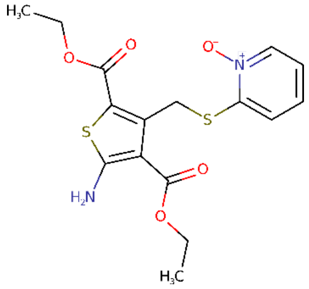	Z18545771	CHEMBL1090045	0.165354	32.5
compound14	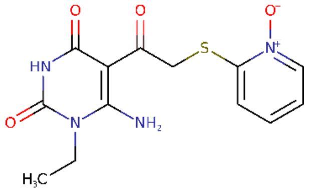	Z91875706	CHEMBL1090045	0.234043	-2
compound15	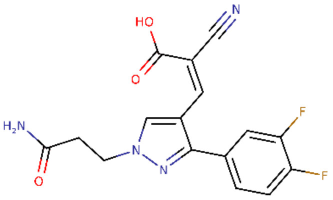	Z56347605	CHEMBL1090045	0.195122	-1.3
compound16	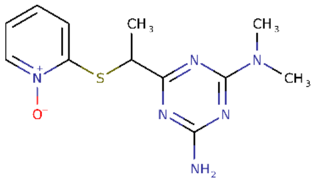	Z193664632	CHEMBL1090045	0.137931	-4.4
compound17	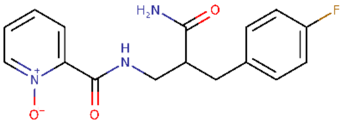	Z910722708	CHEMBL1090045	0.153226	-4.6
compound18	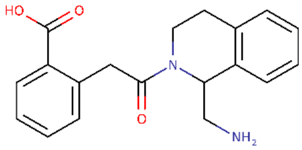	Z276380028 3	CHEMBL1090045	0.204918	-8.2
compound19	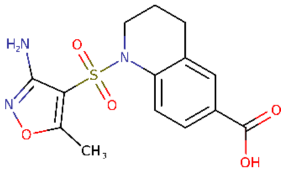	Z243705728 7	CHEMBL1090045	0.256637	-1.1
compound20	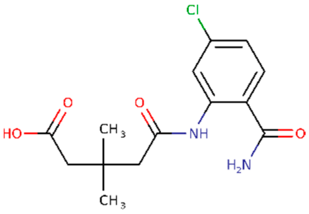	Z369440552	CHEMBL1090045	0.1875	-0.3

ID	Structure	Enamine ID	Reference[1]	Similarity[2]	% Inh.
compound21	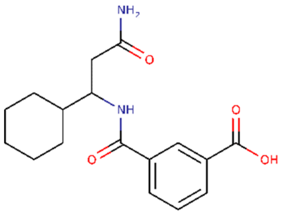	Z1870479217	CHEMBL1090045	0.265487	-1.9
compound22	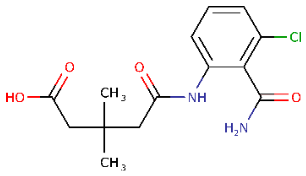	Z1278588990	CHEMBL1090045	0.165217	-4
compound23	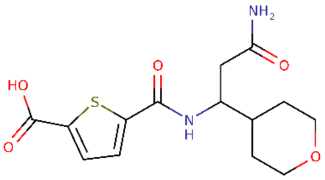	Z1444193603	CHEMBL1090045	0.196581	-3.1
compound24	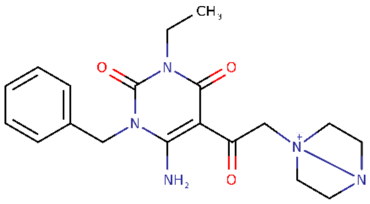	Z48811112	CHEMBL1090045	0.254545	0.8
compound25	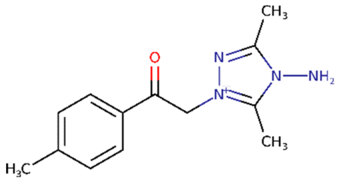	Z56800556	CHEMBL1090045	0.185185	-5.4
compound26	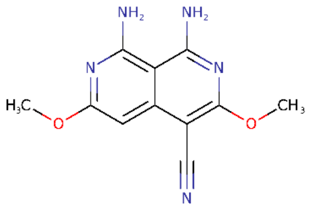	Z56847740	CHEMBL3327225	0.179775	0.9
compound27	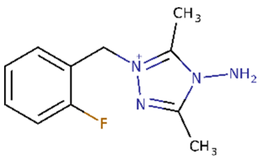	Z56800859	CHEMBL1090045	0.153846	-2.4

[1]Most similar compound to test compound, [2]Morgan fingerprint similarity between test and reference compounds
